# Predictors of the sustainability for an evidence-based eating disorder prevention program delivered by college peer educators

**DOI:** 10.1186/s13012-024-01373-9

**Published:** 2024-07-04

**Authors:** Sarah Kate Bearman, Paul Rohde, Sydney Pauling, Jeff M. Gau, Heather Shaw, Eric Stice

**Affiliations:** 1 Department of Educational Psychology, The University of Texas at Austin, 1912 Speedway, Austin, TX D580078712-0383 USA; 2 Oregon Research Institute, 3800 Sports Way, Springfield, OR 97477 USA; 3 Psychiatry and Behavioral Sciences, 401 Quarry Road, Stanford, CA 94305-5719 USA

**Keywords:** Sustainability, Implementation, Evidence-Based, Prevention, Colleges

## Abstract

**Background:**

Despite ongoing efforts to introduce evidence-based interventions (EBIs) into mental health care settings, little research has focused on the sustainability of EBIs in these settings. College campuses are a natural place to intervene with young adults who are at high risk for mental health disorders, including eating disorders. The current study tested the effect of three levels of implementation support on the sustainability of an evidence-based group eating disorder prevention program, the *Body Project*, delivered by peer educators. We also tested whether intervention, contextual, or implementation process factors predicted sustainability.

**Methods:**

We recruited 63 colleges with peer educator programs and randomly assigned them to (a) receive a 2-day Train-the-Trainer (TTT) training in which peer educators were trained to implement the *Body Project* and supervisors were taught how to train future peer educators (TTT), (b) TTT training plus a technical assistance (TA) workshop (TTT + TA), or (c) TTT plus the TA workshop and quality assurance (QA) consultations over 1-year (TTT + TA + QA). We tested whether implementation support strategies, perceived characteristics of the intervention and attitudes towards evidence-based interventions at baseline and the proportion of completed implementation activities during the implementation year predicted three school-level dichotomous sustainability outcomes (offering *Body Project* groups, training peer educators, training supervisors) over the subsequent two-year sustainability period using logistic regression models.

**Results:**

Implementation support strategies did not significantly predict any sustainability outcomes, although a trend suggested that colleges randomized to the TTT + TA + QA strategy were more likely to train new supervisors (OR = 5.46, 95% CI [0.89–33.38]). Colleges that completed a greater proportion of implementation activities were more likely to offer *Body Project* groups (*OR* = 1.53, 95% CI [1.19–1.98]) and train new peer educators during the sustainability phase (*OR* = 1.39, 95% CI [1.10–1.74]). Perceived positive characteristics of the *Body Project* predicted training new peer educators (*OR* = 18.42, 95% CI [1.48–299.66]), which may be critical for sustainability in routine settings with high provider turnover.

**Conclusions:**

Helping schools complete more implementation activities and increasing the perceived positive characteristics of a prevention program may result in greater sustainment of prevention program implementation.

**Trial Registration:**

This study was preregistered on 12/07/17 with ClinicalTrials.gov, ID NCT03409809, https://clinicaltrials.gov/ct2/show/NCT03409809.

**Supplementary Information:**

The online version contains supplementary material available at 10.1186/s13012-024-01373-9.

Contributions to the literature

- Most evidence-based programs are not sustained after implementation, And few studies have examined program sustainability on college campuses
- College campuses randomly assigned to receive training, technical assistance, and quality assurance for an eating disorder prevention program trended towards training more new supervisors during a 2-year sustainability phase than colleges receiving less support.
- The process of initial implementation and perceived characteristics of the intervention significantly predicted sustainability outcomes.
- The extent to which initial implementation activities are completed may be a particularly robust indicator of sustainability.
- This study highlights potentially malleable predictors of sustainability indicated by theoretical models of implementation.

## Background

Over the last several decades, efforts to develop and test mental health prevention and treatment programs have resulted in an impressive list of evidence-based interventions (EBIs). However, individuals in need of mental health services remain unlikely to receive an EBI. This public health dilemma has prompted increased consideration of factors that promote EBI implementation in the complex settings where most people receive mental health services, including universities and colleges (hereafter, colleges). Implementation involves several processes and outcomes, among them initial adoption, fidelity, feasibility, acceptability, effectiveness, and sustainability [1]. Of these, sustainability—the “extent to which a newly implemented treatment is maintained or institutionalized within a service setting’s ongoing, stable operations” [1] (p70)—has received relatively little attention [2]. Given the high costs associated with initial training and support of EBIs [3, 4], sustainability is an important way to measure return on investment. Additionally, for EBIs to meaningfully reduce the burden of mental illness they must be implemented in a sustained fashion. Few studies have compared different implementation strategies using experimental designs and evaluated their impact on EBI sustainability [2, 5], and even fewer have studied EBI sustainability in college settings [6].

Most young adults attend college [7], making college campuses a natural place to intervene with mental health disorders—especially given the high prevalence of these disorders among college students [8, 9] which has increased following COVID-19 [10]. Prolonged isolation during the pandemic has been linked to various mental health concerns, including body image distress and eating disorders for adolescents and young adults [11, 12]. Eating disorder prevention efforts are particularly well-suited for college settings, since access to treatment services at college mental health clinics is limited [13, 14], and eating disorders, once developed, are often chronic and associated with high levels of distress, medical complications, impairment, and mortality [15, 16]. Due to the low ratio of mental health providers to college students in need, prevention programs that can be provided by college peer educators could dramatically increase access to services.

Among eating disorder prevention programs, the *Body Project* has a robust evidence base [17]. The *Body Project* is an EBI for the prevention of eating disorders wherein adolescent girls/young women with body image concerns collectively critique pursuit of the thin beauty ideal in verbal, written, and behavioral exercises based on cognitive dissonance principles. Prior studies of the *Body Project* with college peer educators [18–22] have demonstrated significant reductions in eating disorder symptoms and future eating disorder onset. Moreover, a recent randomized trial found that the *Body Project* delivered by college peer educators remained effective across varying levels of implementation support [23] and had similar results on other key implementation outcomes (reach, adherence, competence). Although the *Body Project* demonstrated substantial benefits, the factors that predict sustainability are unknown. A mixed-methods naturalistic study of college clinician reactions to the *Body Project* two years after an effectiveness trial found that while 50% of colleges continued to implement the *Body Project* at one-year follow-up, by year 2 only one college (12%) sustained the practice [24].

Most research related to sustainability of EBIs has been in the form of qualitative interviews and retrospective surveys after active implementation efforts [5, 25], alongside conceptual papers. Results of studies set in mental health clinics [26, 27], Veteran’s Affairs hospitals [28], primary care [29] K-12 schools [30, 31] and colleges [24] suggest that numerous factors influence whether an intervention is sustained, including characteristics of the intervention and the practitioners who provide it. This is consistent with theoretical frameworks of sustainability, such as the dynamic sustainability framework (DSF [32]), which proposes that features of the interventions, practice settings, and the broader ecological system affect sustainability both directly and interactively. The DSF further contends that the better the fit between the intervention and the setting, the more likely that the intervention will be maintained^32(p6)^. A unique aspect of the DSF is the expectation that as the practice setting and broader context change dynamically, so too must the intervention to maintain fit. Thus, even interventions that are considered already “optimized” based on efficacy and effectiveness testing may be difficult to sustain amidst ever-changing service settings. Perhaps that is why providers often report modifying interventions when they are sustained [26, 33, 34].

Implementation strategies like provider training, ongoing support, and quality assurance are embedded in conceptual frameworks like the DSF and could impact intervention sustainability. A study of parent–child interaction therapy that randomized 50 mental health organizations to a cascading “train-the-trainer” (TTT) model of training, a learning collaborative model, or a distance learning model found that clinicians in the TTT model implemented the treatment approach with more clients at the 24-month follow-up assessment [35]. Continued reach of the EBI over time is a critical marker of sustainability.

Adequate staffing of individuals who are trained to provide the EBI is another key indicator of a setting’s ability to sustain a practice [36]. Lack of training opportunities following initial implementation has been identified as a barrier to sustainability [37], despite evidence that later generations of providers trained internally can implement EBIs with a comparable level of fidelity as those originally trained by intervention developers [38]. Supervisory support within a setting has also been found to promote adherent practice and client benefit [39, 40]; in contrast, when internal supervisors are not adequately trained in the EBI being offered by their supervisees, working alliance is lessened which may affect service quality [41]. Thus, an organization’s ability to continue to offer an EBI is also affected by their ability to maintain a trained staff of providers and supervisors beyond those initially trained despite the high rates of expected turnover among mental health providers [42], which can undermine sustainability [31]. On college campuses where mental health promotion and prevention efforts are often provided by peer educators [43], sustainability efforts are made more complicated by the transient nature of college students. We were unable to find any studies that measured the extent to which activities like trainings continued after initial EBI implementation, or that examined variables associated with those activities, despite their relevance to an organization’s internal capacity to sustain services.

### The current study

Considerable resources have been invested in the development, testing, and initial implementation of EBIs, but without attention to sustainability their potential for real impact is limited. As noted, college students are a population with a high prevalence of mental health problems, including eating disorders, and often lack access to EBIs. The *Body Project* is a highly effective preventive intervention that can be efficaciously provided by college peer educators, however a rigorous evaluation of its sustainability within college peer educator programs has not been conducted. As part of a larger implementation study [44], we recruited 63 colleges with peer educator programs and randomly assigned them to one of three implementation strategies: Train-the-Trainer (TTT) training, TTT training plus a technical assistance workshop (TTT + TA), and TTT plus the TA workshop and one year of quality assurance (TTT + TA + QA). After one year of active implementation, results indicated that implementation support strategies did not significantly predict primary outcomes including attendance, adherence, competence, and reach. However, adding technical assistance and quality assurance strategies was related to larger reductions in eating disorder risk factors and symptoms. Following active implementation, all participating colleges entered a two-year sustainability phase. The current study compared the impact of different levels of implementation support for the *Body Project* on secondary outcomes related to sustainability.

Our main research questions concerned whether level of implementation support would predict whether *Body Project* groups were conducted during the two-year sustainability phase, and secondarily whether level of implementation support predicted two key ongoing implementation activities: training new peer educators and training new supervisors. Based on main effects reported by Stice et al. [44], we hypothesized that the TTT + TA + QA implementation support strategy would be superior to both the TTT alone and TTT + TA strategies in terms of conducting *Body Project* groups and conducting internal trainings for new peer educators and staff. Although the technical assistance (TA) workshop was intended to enhance the fit of the intervention to each individual context, which has been theorized to result in better sustainability [32], differences between the TTT and TTT + TA strategies during implementation were nonsignificant or small in magnitude. In contrast, the addition of QA consultations, which explicitly focused on managing barriers to implementation and planning for sustainability, resulted in stronger effects during implementation. In alignment with the DSF, the QA consultations permitted progress review, problem-solving of implementation barriers, and sustainability planning, which theoretically would improve the “optimization” of the *Body Project* for long-term sustainment.

Because intervention factors (perceived characteristics of the EBI), provider characteristics (attitudes and beliefs) and implementation process (the completion of implementation activities) have emerged as possible predictors of sustainability in existing qualitative and naturalistic studies of EBT sustainability, we also explored whether provider’s perceived characteristics of the intervention, supervisor attitudes towards the delivery of evidence-based practices, and degree of completion of activities during implementation predicted sustainability outcomes. Because they predicted initial implementation outcomes in a previously published report of the same study [23], we hypothesized that supervisor attitudes towards evidence-based practices and perceived intervention characteristics, and a greater number of completed implementation activities would predict all three sustainability outcomes. All models included covariates related to the organizational context of the colleges since prior research on the *Body Project* found that available resources are related to some implementation outcomes [45], and because theoretical models such as the DSF and others describe these outer and inner settings implementation indicators as influential to sustainability [32, 46].

## Method

### Participants and procedures

US colleges with peer educator programs not already implementing the *Body Project* were contacted to participate. To be included, schools had to have (a) two or more peer education supervisors (college employees who oversee peer educator programming), and (b) eight or more peer educators interested in this training. Sixty-three colleges agreed to participate and were trained. Once the peer educator supervisors and peer educators were trained by project staff, schools recruited undergraduate participants to complete a body acceptance program (which is how the *Body Project* is typically described). Interested female-identifying students were directed to a screening web page to confirm that they had body image concerns (the sole inclusion criterion) and administered the Eating Disorder Diagnostic Scale [44]. No exclusion criteria were recommended. This study’s design and hypotheses were preregistered; see https://clinicaltrials.gov/ct2/show/NCT03409809. The Standards for Reporting Implementation Studies checklist has been included as an additional file. Other details of the study, including student group member characteristics, recruitment procedures, inclusion criteria, and the consideration of sample size are provided by Stice et al. [23] and Rohde et al. [45].

Although student group members were research participants for other aspects of this study, they did not provide any data during the sustainability period. Thus, the present report relies solely on outcome data provided by the peer educators and supervisors supervisors. Supervisors completed an interview and surveys before and after the initial TTT training assessing knowledge and attitudes towards eating disorder prevention, as well as the needs and resources (including the peer education program) on their campus. Supervisors then completed Qualtrics surveys assessing implementation progress at one-year (i.e., end of implementation), two-year, and three-year (i.e., end of Sustainability) follow-ups; the two- and three-year sustainability surveys were completed by one peer education supervisor at each college who had knowledge of the program’s delivery on campus but may not have completed previous assessments (due to staff turnover). Peer educators completed a survey after training assessing their perceptions of the characteristics of the *Body Project*. Neither supervisors nor peer educators received monetary compensation.

### Sustainability outcome measures

*Implementation and Sustainability Progress*. Because we were unable to locate a measure that assesses adoption and implementation of prevention programs, we created the Prevention Implementation Progress Scale (PIPS). We drew general principles for our scale from the Progress Monitoring Instrument (PMI [47]) and the Stages of Implementation Completion measure (SIC [48]). Following the strategies used by these research teams, we delineated the major milestones of the implementation procedure for this prevention program. This measure assesses the following discrete and observable steps in the implementation process: (1) engagement, (2) consideration of feasibility, (3) readiness planning, (4) staff recruitment and training, (5) service initiation, (6) ongoing service provision, (7) adherence monitoring and (8) competency. Most steps were assessed with multiple items that were obtained by a Qualtrics survey (followed up with clarifying emails, if necessary). We used a preliminary version of the PIPS in a previous implementation project with the *Body Project*, where it showed predictive validity; *Body Project* participants in organizations that completed 90% or more versus fewer of the activities in the implementation process showed significantly greater pre-to-post declines in avoidance of life activities due to body image concerns and eating disorder symptoms (M *d* = 0.27). Herein, we report three outcome measures from the PIPS occurring in the two-year sustainability period: (1) whether the school conducted one or more *Body Project* groups (primary outcome), (2) whether the school trained new peer educators to conduct *Body Project* groups, and (3) whether the school trained one or more new peer education supervisors on this EBI.

### Predictor variables

#### Implementation support strategies

Colleges were randomly assigned to receive TTT only (*n* = 21), TTT + TA (*n* = 21), or TTT + TA + QA (*n* = 21), which resulted in 264 *Body Project* groups implemented by 613 peer educators to 1,387 students in the one-year Implementation period following TTT. Peer educators and supervisors in all three support strategy arms were given the tools needed to conduct *Body Project* groups, including the intervention script and access to a facilitator support website (https://sticebodyprojectsupport.weebly.com). Implementation support strategies pairwise contrasts using dummy variables were included in the models described below and include TTT (= 0) vs. TTT + TA (= 1), TTT (= 0) vs. TTT + TA + QA (= 1), and TTT + TA (= 0) vs. TTT + TA + QA (= 1).

#### Train-The-Trainer strategy

All schools received TTT training via an on-campus 2-day TTT training that has been previously validated [21, 22] by an expert trainer. Training included: (1) reviewing the intervention theory, components, and evidence base, (2) roleplay delivery of sessions by teams of peer educators, (3) supervisory feedback from the trainer and supervisors to train peer educators, (4) completion of homework assignments, and (5) reviewing facilitation skills, common problems, assessment, and crisis response plans.

#### Technical assistance strategy

Schools randomized to TTT + TA received an additional ½ day of training to articulate goals, needs, leadership structure, communication, adoption options, and recruitment strategies. The expert trainer met with trainees to assess the practical needs and resources for successful implementation and sustainability. They also answered remaining questions.

#### Quality assurance strategy

Supervisors at schools randomized to TTT + TA + QA were offered monthly consultation calls with their trainer over a 1-year period following the TTT and TA training. These phone or video consultation meetings reviewed progress in conducting groups, problem-solving barriers, planning future implementation events, knowledge sharing, and sustainability planning.

### Intervention, contextual, and implementation process factors

*Perceived Characteristics of the Intervention*. Following TTT training, peer educators and their supervisors completed a survey assessing perceptions of relative advantage, compatibility, complexity, observability, and trialability [49] in an organization. We used the 28-item adoption scale (the Provider Intervention Adoption Scale [50]), modified to refer to the *Body Project*. Response options were on a 5-point scale (1 = strongly disagree, 5 = strongly agree). Exploratory factor analyses identified two factors in the present data. The first factor included 19 items that measured positive perceptions of *Body Project* characteristics (α = 0.96 and 0.93 for peer educators and supervisors, respectively). The second factor included 9 items that measured negative perceptions of *Body Project* characteristics (α = 0.87 and 0.77 for peer educators and supervisors, respectively). All scales were averages across items unless otherwise indicated.

*The Modified Practice Attitudes Scale* (MPAS [51]) is an 8-item measure designed to assess therapists’ attitudes towards evidence-based practices. The eight items use a five-point Likert scale to indicate the extent to which the participant agrees or disagrees with each statement (0 = “not at all” and 4 = “to a very great extent”). Higher scores indicate more favorable attitudes towards evidence-based practices. The MPAS has demonstrated acceptable internal consistency (α = 0.77-0.80) in studies of community mental health providers [52]. A mean score was computed, and higher scores indicate greater support for evidence-based practices (α = 0.77).

*Implementation process factors*. We used 11 indicators of implementation progress collected from the PIPS during the 1-year Implementation period as a potential predictor of greater sustainability. Items addressed the school beginning recruitment for a *Body Project* group, beginning each of up to six groups during implementation, training new peer educators and supervisors in the intervention, and providing structured (observation-based) supervision of peer educator delivery of the *Body Project*. Schools received one point for completion of each activity, with scores ranging from 0–11.

### Covariate adjustments

#### Outer and inner setting implementation indicators

Covariates hypothesized to contribute to the variance of sustainability outcomes are derived from the outer and inner settings domains of the Consolidated Framework for Implementation Research (CFIR [46]). For a longer description, see Rohde et al [53]. At baseline, we evaluated the presence or absence of formal policies related to EBPs, fiscal and other organizational resources for peer educator programs and the student mental health centers, and the mental health needs and resources of students using a standardized qualitative interview conducted with peer educator supervisors prior to the TTT training to assess the following CFIR domains: (1) Available Resources (i.e., *How well-resourced [staffing, time, and budget] is the university?*); (2) Access to Knowledge and Information (i.e., *Does the supervisor feel that they can easily find new information and do they seek it out?*); and (3) Leadership Engagement (i.e., *How supported do supervisors feel by their supervisor at the university?*). Interviews were audio recorded and transcribed, and responses were coded into 3-point quantitative scores per domain, with double coding to ensure acceptable inter-rater reliability: Available resources (0 = not supported in staffing, time budget; 1 = support in one area, 2 = support in 2 + areas); Access to knowledge and information (0 = no access, 1 = one outlet they can use to access information, 2 = materials are available to them from multiple outlets); and Leadership Engagement (0 = minimal/no involvement, 1 = feels support but leader could be more involved, 2 = feels supported).

The Team Climate Inventory [54] was completed by peer educators and supervisors at baseline to measure perceived organizational climate, an inner setting factor. This 14-item survey assesses team Vision (e.g., I am in agreement with the objectives of our peer education program), Participative Safety (e.g., People in the peer education program feel understood and accepted by each other), Task Orientation (e.g., Peer education team members are prepared to question the basis of what the team is doing), and Support for Innovation (e.g., In this peer education team we take the time needed to develop new ideas). Response options were on a 5-point Likert scale of agreement (1 = strongly disagree; 5 = strongly agree). It has shown internal consistency (α = 0.90—0.93) and predictive validity for team innovation and effectiveness [54–56]. For the current study, a total composite mean score was computed, with a higher score indicating greater levels of team climate. Internal consistency was high for peer educators and supervisors (α = 0.96 and 0.95, respectively), and scores were grand mean centered.

### Data analysis

#### Preliminary analyses

We examined the distribution of variables, extent of missing data, summarized the sample of supervisors and peer educators, and examined the distribution and intercorrelations of the predictors.

### Prediction models

We relied on colleges to provide data matching supervisor and peers to groups, however, less than 50% of colleges provided those data. Thus, multilevel modeling of supervisors and peers nested with groups was not possible and we conducted non-nested models with mean supervisor and peer-educator predictors for each school. We examined logistic regression models for dichotomous outcomes that were estimated with a logit link using SPSS (Version 26). All continuous predictor variables were mean-centered. For each outcome, we estimated one model for each of the nine predictors and each model simultaneously adjusted for the four covariates. The likelihood ratio test was used to evaluate model fit. The likelihood ratio test does not always perform well [57] and others have suggested the Hosmer–Lemeshow test [58] as an alternative, however, these require larger sample sizes, with some recommending up to 400 [59]. The Nagelkerke pseudo R^2^ was used to approximate percentage of variance accounted for [60] and was selected over the Cox and Snell pseudo R^2^ because the Cox and Snell pseudo R^2^ value cannot reach 1.0 [61]. Post modeling, we examined for the presence of multicollinearity by inspecting the variance inflation factor (VIF) for model predictors, with values greater than 10.0 indicating the presence of multicollinearity [62]. For effect sizes, we report odds ratios with 95% confidence intervals. Although we did not correct for multiple testing given limited sensitivity with the uncorrected analyses, we computed and report the Benjamini–Hochberg false discovery rate adjusted p-values [63] along with the unadjusted *p*-values in Table 3.

## Results

### Preliminary analyses

Table 1 provides a descriptive summary for the three outcomes. Due to the low endorsement of non-zero categories, we created three dichotomous (Yes/No) outcome measures for the variables. Complete data were available for all 63 colleges.

**Table 1:** Descriptive Summary of Outcome Measures

	TTT (*n* = 21)		TTT + TA (*n* = 21)		TTT + TA + TQ (*n* = 21)	
Number of *Body Project* groups during sustainability (*n*, %)						
0	13	61.9	15	71.4	10	47.6
1	0	0.0	1	4.8	3	14.3
2	1	4.8	0	0.0	1	4.8
3	1	4.8	1	4.8	3	14.3
4	1	4.8	2	9.5	4	19.0
5	1	4.8	0	0.0	0	0.0
6	1	4.8	1	4.8	0	0.0
7	1	4.8	0	0.0	0	0.0
8 or more	2	9.5	1	4.8	0	0.0
Number of new peer educators trained during sustainability (*n*, %)						
0	10	47.6	15	71.4	9	42.9
1	0	0.0	0	0.0	1	4.8
2	0	0.0	0	0.0	2	9.5
3	2	9.5	0	0.0	1	4.8
4	0	0.0	1	4.8	3	14.3
5	2	9.5	0	0.0	2	9.5
6	1	4.8	2	9.5	0	0.0
7	2	9.5	2	9.5	0	0.0
8	0	0.0	0	0.0	0	0.0
9	0	0.0	0	0.0	0	0.0
10 or more	4	19.2	1	4.8	3	4.8
Number of new supervisors trained during sustainability (*n*, %)						
0	17	81.0	18	85.7	13	61.9
1	2	9.5	2	9.5	4	19.0
2	1	4.8	1	4.8	1	4.8
3 or more	1	4.8	0	0.0	3	14.3

We collected data from 232 supervisors and 510 peer educators. On average, there were 4.1 (SD = 2.0, minimum = 1, maximum = 12) supervisors and 10.0 (SD = 3.86, minimum = 3, maximum = 26) peer educators per college. The supervisors were mostly female (93%) and primarily reported their age as between 26 – 30 years of age (23%) followed by 31 – 35 years of age (20%) and 51 years or older (14%). The majority identified as White (74%), followed by Black (8%), Hispanic (7%), Asian (6%) and multi-racial (4%). Peer educators were also mostly female (96%), with a mean age of 21.0 years (*SD* = 3.5) and identified primarily as White (51%), followed by Hispanic (19%), Black (15%), Asian (10%), Native American (3%), and Pacific Islander (2%). A minority of the supervisors (13%) and peer educators (21%) reported a history of eating disorders, and most peer educators (75%) indicated that body image concerns kept them from doing something they enjoyed. We examined whether supervisor or peer educator self-reported demographic characteristics and history of eating disorder or body image concerns were significantly associated with randomized implementation support strategies. No significant associations were found (at *p* < 0.05).

The intercorrelations and descriptive statistics for the 9 predictors are summarized in Table 2. No multicollinearity among the predictors was detected and the largest bivariate correlation was between peer educator reports of positive and negative implementation of the *Body Project* (*r* = -0.56).

**Table 2:** Pearson Correlation Matrix and Descriptive Summary for Predictors

	1	2	3	4	5	6	7	8	9
1. TTT versus TTT + TA	1.00								
2. TTT versus TTT + TA + TQ	^a^	1.00							
3. TTT + TA versus TTT + TA + TQ	^a^	^a^	1.00						
4. PIAS positive, peer educator report	.04 (.785)	.21 (.189)	.16 (.299)	1.00					
5. PIAS negative, peer educator report	-.02 (.883)	.00 (.983)	.03 (.866)	-.56 (< .001)	1.00				
6. PIAS positive, supervisor report	-.16 (.315)	-.03 (.852)	.11 (.488)	.16 (.208)	-.01 (.949)	1.00			
7. PIAS negative, supervisor report	.13 (.427)	-.01 (.993)	-.10 (.544)	-.03 (.791)	.21 (.106)	-.47 (< .001)	1.00		
8. Modified Practice Attitudes Scale	.04 (.803)	-.05 (.776)	-.09 (.583)	.51 (< .001)	-.55 (< .001)	.10 (.442)	-.14 (.275)	1.00	
9. PIPS number of implementation activities	.03 (.843)	.00 (1.00)	-.04 (.812)	.19 (.127)	-.01 (.951)	-.11 (.407)	.09 (.492)	.14 (.276)	1.00
*Mean*	0.50	0.50	0.50	4.39	2.10	4.36	2.12	2.81	4.30
*SD*	0.51	0.51	0.51	0.29	0.35	0.28	0.26	0.21	2.78

*PIAS* Provider Intervention Adoption Scale, *PIPS* Prevention Implementation Progress Scale. TTT coded 0 for first two contrasts and TTT + TA coded 0 for third contrast. Correlation *p*-values presented in parantheses

^a^Cannot be computed because at least one of the variables is a constant

### Prediction models

Annotated parameter estimates from the logistic regression models for the three sustainability outcomes are shown in Table 3 and only include results for the model predictors. See Additional File 1 for results of the full models including parameter estimates for the covariates, and for each model the fit, estimated variance accounted for, and VIF for the predictors and covariates. One variable significantly predicted the primary outcome of a college peer education program conducting one or more *Body Project* groups in the 2-year sustainability period: completion of a greater number of PIPS activities during the first year of implementation (*OR* = 1.48).

**Table 3:** Annotated Results of Logistic Regression Models for College-level Predictors

Outcomes & Predictors	*B*	*SE*	*p-value*	Adj.* p-value*	*OR*	95% *CI*
Conducted a BP groups during sustainability						
TTT versus TTT + TA	-0.54	0.77	.484	.709	0.58	0.13–2.65
TTT versus TTT + TA + TQ	0.26	0.69	.707	.709	1.30	0.33–5.03
TTT + TA versus TTT + TA + TQ	1.11	0.71	.118	.354	3.04	0.76–12.20
PIAS positive, peer educator report	2.37	1.29	.065	.293	10.75	0.86–133.7
PIAS negative, peer educator report	-0.39	0.79	.626	.709	0.68	0.15–3.20
PIAS positive, supervisor report	0.42	1.13	.709	.709	1.53	0.17–14.03
PIAS negative, supervisor report	-0.85	1.17	.469	.709	0.43	0.04–4.26
Modified Practice Attitudes Scale	0.69	1.40	.624	.709	1.99	0.13–31.12
**PIPS number of implementation activities**	**0.43**	**0.13**	**.001**	.009	**1.53**	**1.19–1.98**
Trained new peer educators during sustainability						
TTT versus TTT + TA	-1.20	0.82	.140	.210	0.30	0.06–1.48
TTT versus TTT + TA + TQ	0.05	0.69	.942	.942	1.05	0.27–4.07
TTT + TA versus TTT + TA + TQ	1.10	0.67	.099	.178	3.01	0.81–11.16
**PIAS positive, peer educator report**	**2.91**	**1.29**	**.024**	**.108**	**18.42**	**1.48–299.66**
PIAS negative, peer educator report	-1.43	0.82	.080	.178	0.24	0.05–1.19
PIAS positive, supervisor report	0.38	1.09	.732	.824	1.46	0.17–12.41
PIAS negative, supervisor report	-0.87	1.13	.441	.567	0.42	0.05–3.84
Modified Practice Attitudes Scale	2.41	1.46	.098	.178	11.17	0.64–195.12
**PIPS number of implementation activities**	**0.33**	**0.12**	**.005**	**.045**	**1.39**	**1.10–1.74**
Trained new supervisors during sustainability						
TTT versus TTT + TA	-0.35	0.95	.704	.880	0.70	0.11–4.46
TTT versus TTT + TA + TQ	0.58	0.90	.522	.880	1.78	0.30–10.43
TTT + TA versus TTT + TA + TQ	1.69	0.92	.066	.590	5.46	0.89–33.38
PIAS positive, peer educator report	1.03	1.32	.436	.880	2.80	0.21–37.44
PIAS negative, peer educator report	-0.14	0.96	.880	.880	0.87	0.13–5.67
PIAS positive, supervisor report	1.07	1.37	.434	.880	2.93	0.20–43.26
PIAS negative, supervisor report	-0.21	1.38	.877	.880	0.81	0.50–12.00
Modified Practice Attitudes Scale	0.29	1.60	.854	.880	1.34	0.59–30.67
PIPS number of implementation activities	0.17	0.13	.179	.805	1.19	0.93–1.52

*B* unstandardized beta, *SE* standard error, adj. adjusted *p*-value, *OR* odds ratio, *CI* confidence interval, PIAS Provider Intervention Adoption Scale, PIPS Prevention Implementation Progress Scale. TTT coded 0 for first two contrasts and TTT + TA coded 0 for third contrast. Bolded entries show unadjusted significant predictors at *p* < .05

Two variables significantly predicted whether a new peer educator was trained during sustainability, which included the greater number of PIPS implementation activities (*OR* = 1.39). In addition, higher scores on peer educator positive report of the *Body Project* characteristics were significantly associated with greater odds of whether new peer educators were trained during sustainability (*OR* = 18.42).

No variables were identified that significantly predicted whether a new peer education supervisor was trained during the 2-year sustainability period. The only predictor variable that approached significance (OR = 5.46; *p* = 0.066) was whether the college had received the complete package of implementation support strategies (i.e., ongoing QA in addition to TTT and TA).

## Discussion

The *Body Project* is a group preventive intervention for eating disorders that has proven effectiveness when offered as a peer educator-led service [18–22] and with differing levels of implementation support [23]. The current study is the first to test whether implementation support predicts the sustainability of the *Body Project* on college campuses. Sixty-three college campuses were randomly assigned to three implementation support strategies: TTT only, TTT + TA, and TTT + TA + QA. During a two-year sustainability period, supervisors reported on the number of additional *Body Project* groups conducted, as well as whether they trained new peer educators and supervisors. We hypothesized that schools randomized to the TTT + TA + QA strategy would be more likely to offer *Body Project* groups and would train additional peer educators and supervisors during the sustainability phase. Because theories of sustainability [32] and naturalistic studies of EBI implementation [27] suggest that intervention, provider, and implementation process factors may also influence whether interventions are continued, we also tested whether provider perceived characteristics of the *Body Project*, supervisor attitudes towards evidence-based practices, and the extent to which colleges completed implementation activities in the earlier stages predicted sustainability outcomes. Although the highest level of implementation support approached significance as a predictor of training new supervisors, completion of implementation activities emerged as the strongest predictor of sustainability.

Of the 63 colleges that participated in the study, 38 of them (60%) did not offer any *Body Project* groups following the active year of implementation and reported that no students participated in groups, perhaps in part due to disruptions caused by COVID-19 during follow-up. Among the colleges that did continue to offer groups, the number of groups reported ranged from one to 13, with an average of 4.3 groups representing on average 0.76% (minimum = 0.09, maximum = 4.34) of the eligible female student population. Likewise, most colleges (54%) did not train new peer educators or peer educator supervisors (76%). The finding that only 40% of colleges implemented the *Body Project* during the sustainability phase was not unexpected, as a prior review of EBI implementation efforts noted that programs are rarely sustained in full over time [25]. Indeed, the rates of *Body Project* sustainment compare favorably to the rates of sustainment described in a study of over 1,000 sites using the Universal Stages of Implementation Completion scale, which averaged between 25 to 37%, depending on the definition of sustainment used [64]. Nonetheless, this rate of discontinuation underscores the need for increased attention to the factors that bolster program continuation, particularly those factors that may be malleable.

We hypothesized that level of implementation support might be one malleable factor, however there were no significant differences by implementation support strategies for whether *Body Project* groups were offered or peer educators trained. Colleges that were randomly assigned to receive QA in addition to TTT and TA during the implementation stage appeared to be more likely to train new supervisors during the two-year sustainability period, relative to those who received TTT and TTT + TA, but this was only a trend. The odds ratio (OR = 5.46) corresponds to a moderate-to-large association [65] with a large confidence interval that may reflect the small sample size. Nonetheless, this trend makes intuitive sense given that QA calls specifically included a focus on sustainability planning, which would address continued training of supervisors. Supervisors in the study were trained not only to provide clinical oversight of peer educator-led groups but also to train new peer educators, an activity critical to the longevity of the *Body Project*. In a randomized trial of parent–child interaction therapy training, the training arm in which internal supervisors were trained to provide ongoing training and support resulted in more clients seen at 24 months following active implementation, even though there were no differences between this arm and the others (Learning Collaborative and Distance Education) in number of total clients seen since the initial training [35]. It may be that investing in trainers and supervisors results in more durable services over time, even if it does not initially predict greater reach.

The number of completed activities during the active implementation stage was the most robust predictor of sustainability outcomes. Prior research has shown that completing a greater proportion of activities in the early stages of implementation predicted program start-up in a study of multidimensional treatment foster care in 53 child-welfare serving sites, using different stages of the Stages of Implementation Completion [66]. Our measure of implementation progress likewise measured the proportion of completed activities across the different stages of implementation and we found that greater completion of critical activities during the implementation stage predicted whether groups were offered during the sustainability stage (OR = 1.53), as well as whether new peer educators were trained (OR = 1.39), both small but durable effects [65] that remained significant even after correction for multiple tests. This relationship could have emerged for a variety of reasons. Colleges that are more committed to an EBI may be more likely to complete both implementation activities and those during sustainability. Alternatively, colleges that completed fewer implementation activities may have encountered more challenges, including financial constraints, turnover of leaders or EBI champions, or other emergent priorities [66]. Regardless, completion of implementation activities may be a valuable litmus test for later sustainability and could inform decisions about when to invest resources over time.

Consistent with the DSF [32], peer educators’ perceptions of the characteristics of the *Body Project* following their initial training predicted whether new peer educators were trained (*OR* = 18.42) in the expected direction. This effect was very large in magnitude [65], though it became non-significant after correcting for multiple tests. Peer educators’ perceptions that the *Body Project* offered advantages relative to other programs, was compatible with the setting, was appropriately complex, and offered observable benefits predicted the training of new peer educators. This dovetails with the results of a study by Massey Combs and colleagues [31] in which higher teacher ratings of an intervention’s benefit, compatibility and complexity predicted intervention sustainability. Similarly, a study by Lau et al. [27] found that community therapists’ negative perception of particular EBIs predicted their discontinuation. In contrast, supervisors’ perceptions of the Body Project characteristics and evidence-based practices in general did not significantly predict any sustainability outcomes. In other studies, both provider and supervisor endorsement of EBIs has been suggested to foster sustainment [67–69]. The DSF specifically proposes that the better the fit between the intervention and the setting, the more likely that the intervention will be maintained over time. Peer educators are both the intervention providers and members of the student body for whom the *Body Project* is intended, perhaps lending them particular insight into the extent to which the *Body Project* was an optimal fit with their settings’ needs. For colleges where the *Body Project* wasn’t sustained, it is possible that program modifications could increase the likelihood of sustainment, as the DSF suggests that even programs considered already “optimized” may require adjustment to be maintained [32].

### Limitations

The current study is among very few that have used randomization to test the effects of different implementation support strategies on the sustainability of an evidence-based intervention; even fewer studies have studied the sustainability of prevention interventions on college campuses. Despite this, several limitations should be considered. First, all sustainability outcomes were examined at the college level (*N* = 63) which resulted in less-than-optimal power to detect more than large effects. Although 63 organizations is a respectable sample for dissemination and implementation research projects, it is still a small sample in terms of statistical power. We only had a power of 0.68 to detect medium-sized effects (e.g., OR = 3.47), so we were likely unable to detect clinically meaningful effects that were small to medium in magnitude. Second, the active implementation stage of this study for all colleges took place in early 2020 at the start of the COVID-19 pandemic. This disrupted typical procedures for most college campuses and may have influenced a lack of initial implementation for a sizable number of colleges, 17% of whom did not implement the *Body Project* in the active phase. Perhaps related, most colleges did not engage in any of the sustainability activities examined as outcomes in this study. This resulted in low endorsement of non-zero categories and led us to use dichotomous (Yes/No) outcomes for most sustainability measures rather than continuous, which may have reduced statistical power that was already limited due to the relatively modest number of colleges that participated. In addition, during the sustainability phase, we no longer collected participant data so we cannot assess whether *Body Project* groups remained effective over time or whether there were differences in effectiveness by implementation support strategies. Intervention effectiveness is a critical sustainability outcome, and future studies should consider this alongside the outcomes measured in the current study. Third, the study sample size was small, especially in the context of dichotomous outcomes and logistic regression analyses. The small sample hampered the study in a few statistical ways. We were not able to use more appropriate indicators of model fit (e.g., Hosmer–Lemeshow test), explore potential better modeling options such as robust Poisson regression with estimates of risk ratios [70] or include more covariates adjustments in our prediction models. Finally, this study relied on peer educator supervisor self-reports of all outcomes, which may have biased the results.

## Conclusions

The sustainability of evidence-based interventions has been described as “one of the most significant translational research problems of our time” [71] (p2). Any benefits yielded by the development, testing, adoption, and deployment of interventions are short-lived if practices are ultimately abandoned following the initial stages of implementation. In the current study, 60% of participating colleges did not sustain the *Body Project* after the initial, active implementation stage. Although sustainability rates in this study compared favorably to many EBI implementation efforts [64], even intentional efforts to plan for sustainability with the most intensive implementation support strategy did not significantly increase the likelihood of activities such as continuing to offer groups or training new providers.

It is likely that some of the factors that influence sustainability, such as funding and competing organizational priorities, were beyond the reach of the implementation support interventions in the current study [25]. In a qualitative study by Massatti et al [72], key warning signs that an organization might de-adopt a practice included lack of financial resources and evidence that external agents did not support continued implementation efforts. The current study included such outer and inner setting implementation indicators as covariates [46]; though very few were significant predictors of outcomes in any models (see Additional File 1). Other risks included goodness-of-fit of the intervention with employees’ skills and beliefs and perceptions about ease of implementation [72]. Assessing these risks during implementation, using measures like the Evidence Based Practice Sustainability Index [36], could allow for more personalized approaches to increase sustainability and prevent the de-adoption of EBIs.

The proportion of activities that a college completed during the active implementation stage emerged as the most robust predictor of whether *Body Project* groups were offered during the sustainability stage, as well as whether new peer educators were trained to lead future groups. This aligns with conceptual models that describe implementation as a process of moving through distinct stages in a potentially non-linear and recursive fashion, with each stage interacting with and influencing subsequent stages [73]. Studies of other EBIs in settings different from the current study have also found that the proportion of completed activities in one stage predicted overall success [66]; perhaps this type of progress assessment can be used to guide decision-making about whether an organization has received an adequate dose of support during one stage or whether additional efforts to assist with initial implementation may be required to yield ongoing results.

When implementing EBIs in complex settings, results of the current study suggest that provider positive perceptions of the intervention’s characteristics should be intentionally bolstered during training. Likewise, provider concerns about the relative advantage, compatibility, complexity, and benefit of interventions should be assessed and addressed—perhaps through modifications that increase goodness of fit. Future research should consider whether pre-implementation interventions that promote provider positive perceptions of the intervention might improve sustainability for organizations where baseline attitudes are less positive. Ultimately, sustainment of EBIs in complex service settings is critical to realizing the potential of intervention development, testing, and implementation and supporting meaningful improvements in mental health care.

### Supplementary Information

Additional file 1. Full Covariate Adjusted Models for all Predictors and Outcomes.

Additional file 2.

## Data Availability

The data and study materials are available from Eric Stice (e.g., copies of assessment measures; estice@stanford.edu) or Jeff M. Gau (jeffg@ori.org) for analysis code.

## Abbreviations

EBI: Evidence based intervention
TTT: Train-the-trainer
TA: Technical assistance
QA: Quality assurance
DSF: Dynamic Sustainability Framework
PIPS: Prevention Implementation Progress Scale
CFIR: Consolidated Framework for Intervention Research

## References

[1] Proctor E, Silmere H, Raghavan R, Hovmand P, Aarons G, Bunger A, Griffey R, Hensley M (2011). Outcomes for implementation research: conceptual distinctions, measurement challenges, and research agenda. Administration and policy in mental health and mental health services research.

[2] Shelton RC, Cooper BR, Stirman SW (2018). The sustainability of evidence-based interventions and practices in public health and health care. Annu Rev Public Health.

[3] Eisman AB, Kilbourne AM, Dopp AR, Saldana L, Eisenberg D (2020). Economic evaluation in implementation science: making the business case for implementation strategies. Psychiatry Res.

[4] Pegg SL, Walsh LM, Becker-Haimes EM, Ramirez V, Jensen-Doss A (2021). Money makes the world go ‘round: A qualitative examination of the role funding plays in large-scale implementation and sustainment of youth evidence-based practice. Psychol Serv.

[5] Shoesmith A, Hall A, Wolfenden L, Shelton RC, Powell BJ, Brown H, McCrabb S, Sutherland R, Yoong S, Lane C, Booth D (2021). Barriers and facilitators influencing the sustainment of health behaviour interventions in schools and childcare services: a systematic review. Implement Sci.

[6] Soicher RN, Becker-Blease KA, Bostwick KC (2020). Adapting implementation science for higher education research: the systematic study of implementing evidence-based practices in college classrooms. Cognitive research: principles and implications.

[7] Van Strien T, Frijters JE, Van Staveren WA, Defares PB, Deurenberg P (1986). The predictive validity of the Dutch restrained eating scale. Int J Eat Disord.

[8] Center for Collegiate Mental Health. Center for Collegiate Mental Health 2020 Annual Report. University Park: Center for Collegiate Mental Health; 2021. Publication number STA 21-045.

[9] Twenge JM, Cooper AB, Joiner TE, Duffy ME, Binau SG (2019). Age, period, and cohort trends in mood disorder indicators and suicide-related outcomes in a nationally representative dataset, 2005–2017. J Abnorm Psychol.

[10] Wood CI, Yu Z, Sealy DA, Moss I, Zigbuo-Wenzler E, McFadden C, Landi D, Brace AM (2022). Mental health impacts of the COVID-19 pandemic on college students. J Am Coll Health.

[11] Rodgers RF, Lombardo C, Cerolini S, Franko DL, Omori M, Fuller-Tyszkiewicz M, Linardon J, Courtet P, Guillaume S (2020). The impact of the COVID-19 pandemic on eating disorder risk and symptoms. Int J Eat Disord.

[12] Vitagliano JA, Jhe G, Milliren CE, Lin JA, Spigel R, Freizinger M, Woods ER, Forman SF, Richmond TK (2021). COVID-19 and eating disorder and mental health concerns in patients with eating disorders. J Eat Disord.

[13] Auerbach RP, Alonso J, Axinn WG, Cuijpers P, Ebert DD, Green JG, Hwang I, Kessler RC, Liu H, Mortier P, Nock MK (2016). Mental disorders among college students in the World Health Organization world mental health surveys. Psychol Med.

[14] Bailey RJ, Erekson DM, Cattani K, Jensen D, Simpson DM, Klundt J, Vogeler HA, Schmuck D, Worthen VE, Caldwell Y, Beecher ME (2022). Adapting stepped care: Changes to service delivery format in the context of high demand. Psychol Serv.

[15] Arcelus J, Mitchell AJ, Wales J, Nielsen S (2011). Mortality rates in patients with anorexia nervosa and other eating disorders: a meta-analysis of 36 studies. Arch Gen Psychiatry.

[16] Crow SJ, Peterson CB, Swanson SA, Raymond NC, Specker S, Eckert ED, Mitchell JE (2009). Increased mortality in bulimia nervosa and other eating disorders. Am J Psychiatry.

[17] Stice E, Onipede ZA, Marti CN (2021). A meta-analytic review of trials that tested whether eating disorder prevention programs prevent eating disorder onset. Clin Psychol Rev.

[18] Becker CB, Wilson C, Williams A, Kelly M, McDaniel L, Elmquist J (2010). Peer-facilitated cognitive dissonance versus healthy weight eating disorders prevention: A randomized comparison. Body Image.

[19] Halliwell E, Jarman H, McNamara A, Risdon H, Jankowski G (2015). Dissemination of evidence-based body image interventions: A pilot study into the effectiveness of using undergraduate students as interventionists in secondary schools. Body Image.

[20] Stice E, Marti CN, Rohde P (2013). Prevalence, incidence, impairment, and course of the proposed DSM-5 eating disorder diagnoses in an 8-year prospective community study of young women. J Abnorm Psychol.

[21] Stice E, Rohde P, Shaw H, Gau JM (2017). Clinician-led, peer-led, and internet-delivered dissonance-based eating disorder prevention programs: Acute effectiveness of these delivery modalities. J Consult Clin Psychol.

[22] Stice E, Rohde P, Shaw H, Gau JM (2020). Clinician-led, peer-led, and internet-delivered dissonance-based eating disorder prevention programs: Effectiveness of these delivery modalities through 4-year follow-up. J Consult Clin Psychol.

[23] Stice E, Rohde P, Gau JM, Bearman SK, Shaw H (2023). An experimental test of increasing implementation support for college peer educators delivering an evidence-based prevention program. J Consult Clin Psychol.

[24] Rohde P, Shaw H, Butryn ML, Stice E (2015). Assessing program sustainability in an eating disorder prevention effectiveness trial delivered by college clinicians. Behav Res Ther.

[25] Wiltsey Stirman S, Kimberly J, Cook N, Calloway A, Castro F, Charns M (2012). The sustainability of new programs and innovations: a review of the empirical literature and recommendations for future research. Implement Sci.

[26] Bearman SK, Bailin A, Terry R, Weisz JR (2020). After the study ends: A qualitative study of factors influencing intervention sustainability. Prof Psychol Res Pract.

[27] Lau AS, Lind T, Crawley M, Rodriguez A, Smith A, Brookman-Frazee L (2020). When do therapists stop using evidence-based practices? Findings from a mixed method study on system-driven implementation of multiple EBPs for children. Administration and Policy in Mental Health and Mental Health Services Research.

[28] Mohr DC, Rosen CS, Schnurr PP, Orazem RJ, Noorbaloochi S, Clothier BA, Eftekhari A, Bernardy NC, Chard KM, Crowley JJ, Cook JM (2018). The influence of team functioning and workload on sustainability of trauma-focused evidence-based psychotherapies. Psychiatr Serv.

[29] Berkel C, Rudo-Stern J, Abraczinskas M, Wilson C, Lokey F, Flanigan E, Villamar JA, Dishion TJ, Smith JD (2020). Translating evidence-based parenting programs for primary care: Stakeholder recommendations for sustainable implementation. J Community Psychol.

[30] LoCurto J, Pella J, Chan G, Ginsburg G (2020). School-based clinicians sustained use of a cognitive behavioral treatment for anxiety disorders. Sch Ment Heal.

[31] Combs KM, Buckley PR, Lain MA, Drewelow KM, Urano G, Kerns SE (2022). Influence of classroom-level factors on implementation fidelity during scale-up of evidence-based interventions. Prev Sci.

[32] Chambers DA, Glasgow RE, Stange KC (2013). The dynamic sustainability framework: addressing the paradox of sustainment amid ongoing change. Implement Sci.

[33] Chu BC, Talbott Crocco S, Arnold CC, Brown R, Southam-Gerow MA, Weisz JR (2015). Sustained implementation of cognitive-behavioral therapy for youth anxiety and depression: Long-term effects of structured training and consultation on therapist practice in the field. Prof Psychol Res Pract.

[34] Swain K, Whitley R, McHugo GJ, Drake RE (2010). The sustainability of evidence-based practices in routine mental health agencies. Community Ment Health J.

[35] Jackson CB, Herschell AD, Scudder AT, Hart J, Schaffner KF, Kolko DJ, Mrozowski S (2021). Making implementation last: The impact of training design on the sustainability of an evidence-based treatment in a randomized controlled trial. Administration and Policy in Mental Health and Mental Health Services Research.

[36] Manthey TJ, Goscha R (2013). Conceptual Underpinnings of the Evidence-Based Practice Sustainability Index. Best Pract Ment Health.

[37] Loman SL, Rodriguez BJ, Horner RH (2010). Sustainability of a targeted intervention package: first step to success in Oregon. J Emot Behav Disord.

[38] Buchanan R, Chamberlain P, Price JM, Sprengelmeyer P (2013). Examining the equivalence of fidelity over two generations of KEEP implementation: A preliminary analysis. Child Youth Serv Rev.

[39] Schoenwald SK, Sheidow AJ, Chapman JE (2009). Clinical supervision in treatment transport: effects on adherence and outcomes. J Consult Clin Psychol.

[40] Weisz JR, Ugueto AM, Herren J, Marchette LK, Bearman SK, Lee EH, Thomassin K, Alleyne A, Cheron DM, Tweed JL, Hersh J (2018). When the torch is passed, does the flame still burn? Testing a “train the supervisor” model for the Child STEPs treatment program. J Consult Clin Psychol.

[41] Boyd MR, Park AL, Becker KD, Chorpita BF (2021). The relation between training asymmetry and supervisory working alliance: Implications for the role of supervisors in implementation. Clin Superv.

[42] Adams DR, Williams NJ, Becker-Haimes EM, Skriner L, Shaffer L, DeWitt K, Neimark G, Jones DT, Beidas RS (2019). Therapist financial strain and turnover: interactions with system-level implementation of evidence-based practices. Administration and Policy in Mental Health and Mental Health Services Research.

[43] Newton FB, Ender SC. Students helping students: A guide for peer educators on college campuses. San Francisco: John Wiley & Sons; 2010.

[44] Stice E, Fisher M, Martinez E (2004). Eating disorder diagnostic scale: additional evidence of reliability and validity. Psychol Assess.

[45] Rohde P, Bearman SK, Pauling S, Gau JM, Shaw H, Stice E (2023). Setting and Provider Predictors of Implementation Success for an Eating Disorder Prevention Program Delivered by College Peer Educators. Administration and Policy in Mental Health and Mental Health Services Research.

[46] Damschroder LJ, Aron DC, Keith RE, Kirsh SR, Alexander JA, Lowery JC (2009). Fostering implementation of health services research findings into practice: a consolidated framework for advancing implementation science. Implement Sci.

[47] Bergh AM, Arsalo I, Malan AF, Patrick M, Pattinson RC, Phillips N (2005). Measuring implementation progress in kangaroo mother care. Acta Paediatr.

[48] Chamberlain P, Brown CH, Saldana L (2011). Observational measure of implementation progress in community based settings: the stages of implementation completion (SIC). Implement Sci.

[49] Rogers E. Diffusion of Innovations. 5th ed. New York: Free Press; 2003.

[50] Windsor R, Cleary S, Ramiah K, Clark J, Abroms L, Davis A (2013). The Smoking Cessation and Reduction in Pregnancy Treatment (SCRIPT) Adoption Scale: evaluating the diffusion of a tobacco treatment innovation to a statewide prenatal care program and providers. J Health Commun.

[51] Borntrager CF, Chorpita BF, Higa-McMillan C, Weisz JR (2009). Provider attitudes toward evidence-based practices: are the concerns with the evidence or with the manuals?. Psychiatr Serv.

[52] Burgess AM, Okamura KH, Izmirian SC, Higa-McMillan CK, Shimabukuro S, Nakamura BJ (2017). Therapist attitudes towards evidence-based practice: A joint factor analysis. J Behav Health Serv Res.

[53] Rohde P, Bearman SK, Pauling S, Gau JM, Shaw H, Stice E (2023). Setting and Provider Predictors of Implementation Success for an Eating Disorder Prevention Program Delivered by College Peer Educators. Administration and Policy in Mental Health and Mental Health Services Research.

[54] Kivimaki M, Elovainio M (1999). A short version of the Team Climate Inventory: Development and psychometric properties. J Occup Organ Psychol.

[55] Loo R, Loewen P (2002). A confirmatory factor-analytic and psychometric examination of the team climate inventory: full and short versions. Small Group Research.

[56] Anderson N, West MA (1996). The Team Climate Inventory: Development of the TCI and its applications in teambuilding for innovativeness. Eur J Work Organ Psy.

[57] McCullagh P (1985). On the asymptotic distribution of Pearson's statistic in linear exponential-family models. International Statistical Review/Revue Internationale de Statistique.

[58] Hosmer DW, Lemesbow S (1980). Goodness of fit tests for the multiple logistic regression model. Communications in statistics-Theory and Methods.

[59] Allison PD (2014). Measures of fit for logistic regression. In Proceedings of the SAS global forum 2014 conference.

[60] Nagelkerke NJ (1991). A note on a general definition of the coefficient of determination. Biometrika..

[61] Cox DR. Analysis of binary data. New York: Routledge; 2018.

[62] Pallant, J., 2020. SPSS survival manual: A step by step guide to data analysis using IBM SPSS. London: Routledge; 2020.

[63] Benjamini Y, Hochberg Y (1995). Controlling the false discovery rate: a practical and powerful approach to multiple testing. J Roy Stat Soc: Ser B (Methodol).

[64] Wong DR, Schaper H, Saldana L (2022). Rates of sustainment in the Universal Stages of Implementation Completion. Implementation Science Communications.

[65] Chen H, Cohen P, Chen S. How big is a big odds ratio? Interpreting the magnitudes of odds ratios in epidemiological studies. Communications in Statistics—simulation and Computation®. 2010;39(4):860–4. 10.1080/03610911003650383.

[66] Saldana L, Chamberlain P, Wang W, Hendricks BC (2012). Predicting program start-up using the stages of implementation measure. Administration and Policy in Mental Health and Mental Health Services Research.

[67] Horwitz SM, Lewis K, Gleacher A, Wang N, Bradbury DM, Ray-LaBatt M, Hoagwood KE (2019). Sustainability of an evidence-based practice in community mental health agencies serving children. Psychiatr Serv.

[68] Ryba MM, Lo SB, Andersen BL (2021). Sustainability of a biobehavioral intervention implemented by therapists and sustainment in community settings. Translational Behavioral Medicine.

[69] Solberg LI, Brekke ML, Fazio CJ, Fowles J, Jacobsen DN, Kottke TE, Mosser G, O’Connor PJ, Ohnsorg KA, Rolnick SJ (2000). Lessons from experienced guideline implementers: attend to many factors and use multiple strategies. Jt Comm J Qual Improv.

[70] Chen W, Qian L, Shi J, Franklin M (2018). Comparing performance between log-binomial and robust Poisson regression models for estimating risk ratios under model misspecification. BMC Med Res Methodol.

[71] Proctor E, Luke D, Calhoun A, McMillen C, Brownson R, McCrary S, Padek M (2015). Sustainability of evidence-based healthcare: research agenda, methodological advances, and infrastructure support. Implement Sci.

[72] Massatti RR, Sweeney HA, Panzano PC, Roth D (2008). The de-adoption of innovative mental health practices (IMHP): Why organizations choose not to sustain an IMHP. Administration and Policy in Mental Health and Mental Health Services Research.

[73] Fixsen DL, Blase KA, Naoom SF, Van Dyke M, Wallace F (2009). Implementation: The missing link between research and practice. NIRN implementation brief.

