# Supplementary material for: Predictors of the sustainability for an evidence-based eating disorder prevention program delivered by college peer educators
Source: Implement Sci. 2024 Jul 4;19:47. doi: 10.1186/s13012-024-01373-9 (PMC11225113; doi:10.1186/s13012-024-01373-9)
Supplement: Supplementary file 1 — Additional file 1. Full Covariate Adjusted Models for all Predictors and Outcomes. [file 13012_2024_1373_MOESM1_ESM.docx]

**Full Covariate Adjusted Models for all Predictors and Outcomes**

Models 1-9 show results from the multivariable logistic regression models predicting whether a Body Project group was conducted during sustainability. The first line in each table is the predictor followed by four covariate adjustments. In the notes section, the likelihood ratio chi-square test is provided as an indicator of model fit and the Nagelkerke pseudo R^2^ provides an estimate of the percent of variance account for.

Model 1

| *Predictor* and Covariates | *B* | *SE* | *p-value* | *OR* | 95% *CI* | VIF |
| --- | --- | --- | --- | --- | --- | --- |
| *TTT versus TTT+TA* | -0.54 | 0.77 | .484 | 0.58 | 0.13-2.65 | 1.12 |
| Available resources | -0.27 | 0.51 | .594 | 0.76 | 0.28-2.08 | 1.20 |
| Access to knowledge | 1.89 | 1.00 | .059 | 6.61 | 0.93-46.76 | 1.16 |
| Leader engagement | -0.76 | 0.54 | .161 | 0.47 | 0.16-1.35 | 1.32 |
| Team climate | 0.80 | 1.39 | .564 | 2.23 | 0.15-33.64 | 1.11 |
| Notes. *B* = unstandardized beta, *SE* = standard error, *OR* = odds ratio, *CI* = confidence interval, VIF = variance inflation factor. TTT coded 0. Likelihood ratio test χ^2^ = 6.99, *p* = .221; Nagelkerke pseudo *R*^2^ = .21 | | | | | | |

Model 2

| *Predictor* and Covariates | *B* | *SE* | *p-value* | *OR* | 95% *CI* | VIF |
| --- | --- | --- | --- | --- | --- | --- |
| *TTT versus TTT+TA+TQ* | 0.26 | 0.69 | .707 | 1.30 | 0.33-5.03 | 1.06 |
| Available resources | -1.11 | 0.59 | .059 | 0.33 | 0.10-1.04 | 1.18 |
| Access to knowledge | 0.67 | 0.79 | .399 | 1.95 | 0.41-9.13 | 1.22 |
| Leader engagement | -0.17 | 0.49 | .729 | 0.84 | 0.32-2.20 | 1.31 |
| Team climate | -0.26 | 1.17 | .825 | 0.77 | 0.08-7.69 | 1.11 |
| Notes. *B* = unstandardized beta, *SE* = standard error, *OR* = odds ratio, *CI* = confidence interval, VIF = variance inflation factor. TTT coded 0. Likelihood ratio test χ^2^ = 5.63, *p* = .343; Nagelkerke pseudo *R*^2^ = .17 | | | | | | |

Model 3

| *Predictor* and Covariates | *B* | *SE* | *p-value* | *OR* | 95% *CI* | VIF |
| --- | --- | --- | --- | --- | --- | --- |
| *TTT+TA versus TTT+TA+TQ* | 1.11 | 0.71 | .118 | 3.04 | 0.76-12.20 | 1.02 |
| Available resources | -0.67 | 0.53 | .207 | 0.51 | 0.18-1.45 | 1.34 |
| Access to knowledge | 0.52 | 0.73 | .474 | 1.69 | 0.40-7.09 | 1.25 |
| Leader engagement | -0.36 | 0.46 | .469 | 0.72 | 0.29-1.77 | 1.27 |
| Team climate | -0.20 | 1.26 | .872 | 0.82 | 0.07-9.63 | 1.06 |
| Notes. *B* = unstandardized beta, *SE* = standard error, *OR* = odds ratio, *CI* = confidence interval, VIF = variance inflation factor. TTT+TA coded 0. Likelihood ratio test χ^2^ = 5.13, *p* = .400; Nagelkerke pseudo *R*^2^ = .16 | | | | | | |

Model 4

| *Predictor* and Covariates | *B* | *SE* | *p-value* | *OR* | 95% *CI* | VIF |
| --- | --- | --- | --- | --- | --- | --- |
| *PIAS positive, peer educator report* | 2.37 | 1.29 | .065 | 10.75 | 0.86-133.7 | 1.04 |
| Available resources | -0.71 | 0.43 | .100 | 0.49 | 0.21-1.15 | 1.19 |
| Access to knowledge | 0.80 | 0.65 | .221 | 2.22 | 0.62-7.96 | 1.19 |
| Leader engagement | -0.26 | 0.39 | .510 | 0.77 | 0.36-1.66 | 1.32 |
| Team climate | -0.11 | 1.00 | .909 | 0.89 | 0.13-6.37 | 1.04 |
| Notes. *B* = unstandardized beta, *SE* = standard error, *OR* = odds ratio, *CI* = confidence interval, VIF = variance inflation factor, PIAS = Provider Intervention Adoption Scale. Likelihood ratio test χ^2^ = 9.15, *p* = .103; Nagelkerke pseudo *R*^2^ = .19 | | | | | | |

Model 5

| *Predictor* and Covariates | *B* | *SE* | *p-value* | *OR* | 95% *CI* | VIF |
| --- | --- | --- | --- | --- | --- | --- |
| *PIAS negative, peer educator report* | -0.39 | 0.79 | .626 | 0.68 | 0.15-3.20 | 1.05 |
| Available resources | -0.59 | 0.41 | .146 | 0.55 | 0.25-1.23 | 1.19 |
| Access to knowledge | 0.87 | 0.64 | .178 | 2.38 | 0.68-8.40 | 1.18 |
| Leader engagement | -0.34 | 0.39 | .388 | 0.71 | 0.33-1.54 | 1.35 |
| Team climate | 0.12 | 0.95 | .903 | 1.12 | 0.17-7.28 | 1.04 |
| Notes. *B* = unstandardized beta, *SE* = standard error, *OR* = odds ratio, *CI* = confidence interval, VIF = variance inflation factor, PIAS = Provider Intervention Adoption Scale. Likelihood ratio test χ^2^ = 5.19, *p* = .393; Nagelkerke pseudo *R*^2^ = .11 | | | | | | |

Model 6

| *Predictor* and Covariates | *B* | *SE* | *p-value* | *OR* | 95% *CI* | VIF |
| --- | --- | --- | --- | --- | --- | --- |
| *PIAS positive, supervisor report* | 0.42 | 1.13 | .709 | 1.53 | 0.17-14.03 | 1.37 |
| Available resources | -0.60 | 0.41 | .144 | 0.55 | 0.24-1.23 | 1.22 |
| Access to knowledge | 0.94 | 0.65 | .148 | 2.56 | 0.72-9.09 | 1.22 |
| Leader engagement | -0.39 | 0.39 | .313 | 0.68 | 0.32-1.45 | 1.31 |
| Team climate | -0.11 | 1.08 | .920 | 0.90 | 0.11-7.47 | 1.33 |
| Notes. *B* = unstandardized beta, *SE* = standard error, *OR* = odds ratio, *CI* = confidence interval, VIF = variance inflation factor, PIAS = Provider Intervention Adoption Scale. Likelihood ratio test χ^2^ = 5.09, *p* = .405; Nagelkerke pseudo *R*^2^ = .11 | | | | | | |

Model 7

| *Predictor* and Covariates | *B* | *SE* | *p-value* | *OR* | 95% *CI* | VIF |
| --- | --- | --- | --- | --- | --- | --- |
| *PIAS negative, supervisor report* | -0.85 | 1.17 | .469 | 0.43 | 0.04-4.26 | 1.06 |
| Available resources | -0.57 | 0.41 | .159 | 0.56 | 0.25-1.25 | 1.18 |
| Access to knowledge | 0.96 | 0.66 | .145 | 2.61 | 0.72-9.45 | 1.18 |
| Leader engagement | -0.43 | 0.40 | .281 | 0.65 | 0.30-1.41 | 1.31 |
| Team climate | -0.07 | 0.96 | .946 | 0.94 | 0.14-6.15 | 1.07 |
| Notes. *B* = unstandardized beta, *SE* = standard error, *OR* = odds ratio, *CI* = confidence interval, VIF = variance inflation factor, PIAS = Provider Intervention Adoption Scale. Likelihood ratio test χ^2^ = 5.49, *p* = .359; Nagelkerke pseudo *R*^2^ = .12 | | | | | | |

Model 8

| *Predictor* and Covariates | *B* | *SE* | *p-value* | *OR* | 95% *CI* | VIF |
| --- | --- | --- | --- | --- | --- | --- |
| *Modified Practice Attitudes Scale* | 0.69 | 1.40 | .624 | 1.99 | 0.13-31.12 | 1.17 |
| Available resources | -0.58 | 0.41 | .153 | 0.56 | 0.25-1.24 | 1.18 |
| Access to knowledge | 0.86 | 0.65 | .182 | 2.37 | 0.67-8.45 | 1.20 |
| Leader engagement | -0.32 | 0.41 | .434 | 0.73 | 0.33-1.61 | 1.44 |
| Team climate | 0.01 | 0.97 | .989 | 1.01 | 0.15-6.72 | 1.06 |
| Notes. *B* = unstandardized beta, *SE* = standard error, *OR* = odds ratio, *CI* = confidence interval, VIF = variance inflation factor. Likelihood ratio test χ^2^ = 5.20, *p* = .393; Nagelkerke pseudo *R*^2^ = .11 | | | | | | |

Model 9

| *Predictor* and Covariates | *B* | *SE* | *p-value* | *OR* | 95% *CI* | VIF |
| --- | --- | --- | --- | --- | --- | --- |
| *PIPS number of implementation activities* | 0.43 | 0.13 | .001 | 1.53 | 1.19-1.98 | 1.09 |
| Available resources | -0.64 | 0.48 | .183 | 0.53 | 0.21-1.35 | 1.19 |
| Access to knowledge | 0.61 | 0.67 | .357 | 1.85 | 0.50-6.82 | 1.19 |
| Leader engagement | -0.31 | 0.44 | .471 | 0.73 | 0.31-1.72 | 1.30 |
| Team climate | -0.93 | 1.16 | .422 | 0.39 | 0.40-3.87 | 1.09 |
| Notes. *B* = unstandardized beta, *SE* = standard error, *OR* = odds ratio, *CI* = confidence interval, VIF = variance inflation factor, PIPS = Prevention Implementation Progress Scale. Likelihood ratio test χ^2^ = 18.83, *p* = .002; Nagelkerke pseudo *R*^2^ = .37 | | | | | | |

Models 10-18 show results from the multivariable logistic regression models predicting whether a new peer educator was trained during sustainability. The first line in each table is the predictor followed by four covariate adjustments. In the notes section, the likelihood ratio chi-square test is provided as an indicator of model fit and the Nagelkerke pseudo R^2^ provides an estimate of the percent of variance account for.

Model 10

| *Predictor* and Covariates | *B* | *SE* | *p-value* | *OR* | 95% *CI* | VIF |
| --- | --- | --- | --- | --- | --- | --- |
| *TTT versus TTT+TA* | -1.20 | 0.82 | .140 | 0.30 | 0.06-1.48 | 1.12 |
| Available resources | 0.27 | 0.56 | .632 | 1.30 | 0.44-3.87 | 1.20 |
| Access to knowledge | 2.29 | 1.06 | .030 | 9.89 | 1.24-78.71 | 1.16 |
| Leader engagement | -1.57 | 0.63 | .013 | 0.21 | 0.06-0.71 | 1.33 |
| Team climate | 1.31 | 1.51 | .385 | 3.71 | 0.19-71.47 | 1.10 |
| Notes. *B* = unstandardized beta, *SE* = standard error, *OR* = odds ratio, *CI* = confidence interval, VIF = variance inflation factor. TTT coded 0. Likelihood ratio test χ^2^ = 14.90, *p* = .011; Nagelkerke pseudo *R*^2^ = .40 | | | | | | |

Model 11

| *Predictor* and Covariates | *B* | *SE* | *p-value* | *OR* | 95% *CI* | VIF |
| --- | --- | --- | --- | --- | --- | --- |
| *TTT versus TTT+TA+TQ* | 0.05 | 0.69 | .942 | 1.05 | 0.27-4.07 | 1.06 |
| Available resources | -0.78 | 0.55 | .153 | 0.46 | 0.16-1.34 | 1.18 |
| Access to knowledge | 0.44 | 0.76 | .563 | 1.55 | 0.35-6.88 | 1.22 |
| Leader engagement | -0.28 | 0.48 | .559 | 0.76 | 0.30-1.94 | 1.31 |
| Team climate | 1.43 | 1.21 | .238 | 4.18 | 0.39-44.90 | 1.14 |
| Notes. *B* = unstandardized beta, *SE* = standard error, *OR* = odds ratio, *CI* = confidence interval, VIF = variance inflation factor. TTT coded 0. Likelihood ratio test χ^2^ = 5.07, *p* = .407; Nagelkerke pseudo *R*^2^ = .16 | | | | | | |

Model 12

| *Predictor* and Covariates | *B* | *SE* | *p-value* | *OR* | 95% *CI* | VIF |
| --- | --- | --- | --- | --- | --- | --- |
| *TTT+TA versus TTT+TA+TQ* | 1.10 | 0.67 | .099 | 3.01 | 0.81-11.16 | 1.02 |
| Available resources | 0.15 | 0.51 | .763 | 1.17 | 0.43-3.16 | 1.34 |
| Access to knowledge | 0.11 | 0.69 | .873 | 1.12 | 0.29-4.31 | 1.25 |
| Leader engagement | -0.21 | 0.46 | .649 | 0.81 | 0.33-1.99 | 1.28 |
| Team climate | -0.05 | 1.20 | .968 | 0.95 | 0.09-10.00 | 1.06 |
| Notes. *B* = unstandardized beta, *SE* = standard error, *OR* = odds ratio, *CI* = confidence interval, VIF = variance inflation factor. TTT+TA coded 0. Likelihood ratio test χ^2^ = 3.24, *p* = .663; Nagelkerke pseudo *R*^2^ = .10 | | | | | | |

Model 13

| *Predictor* and Covariates | *B* | *SE* | *p-value* | *OR* | 95% *CI* | VIF |
| --- | --- | --- | --- | --- | --- | --- |
| *PIAS positive, peer educator report* | 2.91 | 1.29 | .024 | 18.42 | 1.48-229.6 | 1.04 |
| Available resources | -0.15 | 0.42 | .720 | 0.86 | 0.38-1.96 | 1.19 |
| Access to knowledge | 0.63 | 0.63 | .316 | 1.87 | 0.55-3.37 | 1.19 |
| Leader engagement | -0.43 | 0.39 | .272 | 0.65 | 0.30-1.40 | 1.32 |
| Team climate | 0.86 | 1.03 | .406 | 2.35 | 0.31-17.68 | 1.04 |
| Notes. *B* = unstandardized beta, *SE* = standard error, *OR* = odds ratio, *CI* = confidence interval, VIF = variance inflation factor, PIAS = Provider Intervention Adoption Scale. Likelihood ratio test χ^2^ = 11.46, *p* = .043; Nagelkerke pseudo *R*^2^ = .23 | | | | | | |

Model 14

| *Predictor* and Covariates | *B* | *SE* | *p-value* | *OR* | 95% *CI* | VIF |
| --- | --- | --- | --- | --- | --- | --- |
| *PIAS negative, peer educator report* | -1.43 | 0.82 | .080 | 0.24 | 0.05-1.19 | 1.05 |
| Available resources | -0.09 | 0.40 | .816 | 0.91 | 0.42-2.00 | 1.19 |
| Access to knowledge | 0.72 | 0.64 | .255 | 2.06 | 0.59-7.17 | 1.18 |
| Leader engagement | -0.44 | 0.40 | .268 | 0.64 | 0.30-1.40 | 1.35 |
| Team climate | 1.18 | 1.00 | .239 | 3.26 | 0.46-23.31 | 1.04 |
| Notes. *B* = unstandardized beta, *SE* = standard error, *OR* = odds ratio, *CI* = confidence interval, VIF = variance inflation factor, PIAS = Provider Intervention Adoption Scale. Likelihood ratio test χ^2^ = 8.09, *p* = .152; Nagelkerke pseudo *R*^2^ = .16 | | | | | | |

Model 15

| *Predictor* and Covariates | *B* | *SE* | *p-value* | *OR* | 95% *CI* | VIF |
| --- | --- | --- | --- | --- | --- | --- |
| *PIAS positive, supervisor report* | 0.38 | 1.09 | .732 | 1.46 | 0.17-12.41 | 1.37 |
| Available resources | -0.05 | 0.40 | .899 | 0.95 | 0.44-2.07 | 1.22 |
| Access to knowledge | 0.85 | 0.63 | .174 | 2.34 | 0.69-7.97 | 1.22 |
| Leader engagement | -0.58 | 0.39 | .138 | 0.56 | 0.26-1.20 | 1.31 |
| Team climate | 0.82 | 1.06 | .441 | 2.26 | 0.28-18.02 | 1.33 |
| Notes. *B* = unstandardized beta, *SE* = standard error, *OR* = odds ratio, *CI* = confidence interval, VIF = variance inflation factor, PIAS = Provider Intervention Adoption Scale. Likelihood ratio test χ^2^ = 4.88, *p* = .431; Nagelkerke pseudo *R*^2^ = ..10 | | | | | | |

Model 16

| *Predictor* and Covariates | *B* | *SE* | *p-value* | *OR* | 95% *CI* | VIF |
| --- | --- | --- | --- | --- | --- | --- |
| *PIAS negative, supervisor report* | -0.87 | 1.13 | .441 | 0.42 | 0.05-3.84 | 1.06 |
| Available resources | -0.02 | 0.39 | .968 | 0.98 | 0.46-2.12 | 1.18 |
| Access to knowledge | 0.90 | 0.64 | .162 | 2.45 | 0.70-8.57 | 1.18 |
| Leader engagement | -0.61 | 0.39 | .120 | 0.54 | 0.25-1.17 | 1.32 |
| Team climate | 0.84 | 0.95 | .377 | 2.32 | 0.36-15.06 | 1.07 |
| Notes. *B* = unstandardized beta, *SE* = standard error, *OR* = odds ratio, *CI* = confidence interval, VIF = variance inflation factor, PIAS = Provider Intervention Adoption Scale. Likelihood ratio test χ^2^ = 5.37, *p* = .373; Nagelkerke pseudo *R*^2^ = .11 | | | | | | |

Model 17

| *Predictor* and Covariates | *B* | *SE* | *p-value* | *OR* | 95% *CI* | VIF |
| --- | --- | --- | --- | --- | --- | --- |
| *Modified Practice Attitudes Scale* | 2.41 | 1.46 | .098 | 11.17 | 0.64-195.1 | 1.17 |
| Available resources | -0.04 | 0.40 | .927 | 0.96 | 0.44-2.12 | 1.18 |
| Access to knowledge | 0.72 | 0.65 | .274 | 2.05 | 0.57-7.37 | 1.20 |
| Leader engagement | -0.37 | 0.41 | .369 | 0.69 | 0.31-1.54 | 1.44 |
| Team climate | 0.81 | 0.98 | .408 | 2.25 | 0.33-15.40 | 1.06 |
| Notes. *B* = unstandardized beta, *SE* = standard error, *OR* = odds ratio, *CI* = confidence interval, VIF = variance inflation factor. Likelihood ratio test χ^2^ = 7.67, *p* = .175; Nagelkerke pseudo *R*^2^ = .16 | | | | | | |

Model 18

| *Predictor* and Covariates | *B* | *SE* | *p-value* | *OR* | 95% *CI* | VIF |
| --- | --- | --- | --- | --- | --- | --- |
| *PIPS number of implementation activities* | 0.33 | 0.12 | .005 | 1.39 | 1.10-1.74 | 1.09 |
| Available resources | 0.07 | 0.44 | .872 | 1.07 | 0.54-2.54 | 1.19 |
| Access to knowledge | 0.53 | 0.62 | .394 | 1.70 | 0.50-5.73 | 1.19 |
| Leader engagement | -0.55 | 0.42 | .182 | 0.58 | 0.26-1.30 | 1.30 |
| Team climate | 0.47 | 1.05 | .654 | 1.60 | 0.21-12.52 | 1.09 |
| Notes. *B* = unstandardized beta, *SE* = standard error, *OR* = odds ratio, *CI* = confidence interval, VIF = variance inflation factor, PIPS = Prevention Implementation Progress Scale. Likelihood ratio test χ^2^ = 14.14, *p* = .015; Nagelkerke pseudo *R*^2^ = .27 | | | | | | |

Models 19-27 show results from the multivariable logistic regression models predicting whether a supervisor was trained during sustainability. The first line in each table is the predictor followed by four covariate adjustments. In the notes section, the likelihood ratio chi-square test is provided as an indicator of model fit and the Nagelkerke pseudo R^2^ provides an estimate of the percent of variance account for.

Model 19

| *Predictor* and Covariates | *B* | *SE* | *p-value* | *OR* | 95% *CI* | VIF |
| --- | --- | --- | --- | --- | --- | --- |
| *TTT versus TTT+TA* | -0.35 | 0.95 | .704 | 0.70 | 0.11-4.46 | 1.12 |
| Available resources | 0.43 | 0.66 | .514 | 1.54 | 0.42-5.59 | 1.20 |
| Access to knowledge | -0.13 | 0.85 | .883 | 0.88 | 0.17-4.69 | 1.16 |
| Leader engagement | -1.03 | 0.63 | .104 | 0.36 | 0.10-1.24 | 1.33 |
| Team climate | 0.13 | 1.61 | .935 | 1.14 | 0.05-26.71 | 1.11 |
| Notes. *B* = unstandardized beta, *SE* = standard error, *OR* = odds ratio, *CI* = confidence interval, VIF = variance inflation factor. TTT coded 0. Likelihood ratio test χ^2^ = 3.61, *p* = .607; Nagelkerke pseudo *R*^2^ = .14 | | | | | | |

Model 20

| *Predictor* and Covariates | *B* | *SE* | *p-value* | *OR* | 95% *CI* | VIF |
| --- | --- | --- | --- | --- | --- | --- |
| *TTT versus TTT+TA+TQ* | 0.58 | 0.90 | .522 | 1.78 | 0.30-10.43 | 1.06 |
| Available resources | -1.99 | 0.87 | .022 | 0.14 | 0.03-0.75 | 1.18 |
| Access to knowledge | 0.14 | 0.96 | .886 | 1.15 | 0.18-7.52 | 1.22 |
| Leader engagement | -0.31 | 0.62 | .622 | 0.74 | 0.22-2.49 | 1.31 |
| Team climate | -0.24 | 1.62 | .883 | 0.79 | 0.03-18.74 | 1.11 |
| Notes. *B* = unstandardized beta, *SE* = standard error, *OR* = odds ratio, *CI* = confidence interval, VIF = variance inflation factor. TTT coded 0. Likelihood ratio test χ^2^ = 12.29, *p* = .031; Nagelkerke pseudo *R*^2^ = .38 | | | | | | |

Model 21

| *Predictor* and Covariates | *B* | *SE* | *p-value* | *OR* | 95% *CI* | VIF |
| --- | --- | --- | --- | --- | --- | --- |
| *TTT+TA versus TTT+TA+TQ* | 1.69 | 0.92 | .066 | 5.46 | 0.89-33.38 | 1.02 |
| Available resources | -0.50 | 0.69 | .465 | 0.60 | 0.16-2.34 | 1.34 |
| Access to knowledge | -0.64 | 0.78 | .412 | 0.53 | 0.11-2.44 | 1.25 |
| Leader engagement | -0.81 | 0.57 | .155 | 0.44 | 0.14-1.36 | 1.28 |
| Team climate | -0.19 | 1.53 | .903 | 0.83 | 0.04-16.60 | 1.06 |
| Notes. *B* = unstandardized beta, *SE* = standard error, *OR* = odds ratio, *CI* = confidence interval, VIF = variance inflation factor. TTT+TA coded 0. Likelihood ratio test χ^2^ = 9.20, *p* = .102; Nagelkerke pseudo *R*^2^ = .30 | | | | | | |

Model 22

| *Predictor* and Covariates | *B* | *SE* | *p-value* | *OR* | 95% *CI* | VIF |
| --- | --- | --- | --- | --- | --- | --- |
| *PIAS positive, peer educator report* | 1.03 | 1.32 | .436 | 2.80 | 0.21-37.44 | 1.04 |
| Available resources | -0.53 | 0.48 | .268 | 0.59 | 0.23-1.50 | 1.19 |
| Access to knowledge | -0.26 | 0.62 | .678 | 0.77 | 0.23-2.60 | 1.19 |
| Leader engagement | -0.61 | 0.45 | .182 | 0.55 | 0.23-1.33 | 1.32 |
| Team climate | -0.46 | 1.14 | .685 | 0.63 | 0.07-5.88 | 1.04 |
| Notes. *B* = unstandardized beta, *SE* = standard error, *OR* = odds ratio, *CI* = confidence interval, VIF = variance inflation factor, PIAS = Provider Intervention Adoption Scale. Likelihood ratio test χ^2^ = 6.80, *p* = .236; Nagelkerke pseudo *R*^2^ = .16 | | | | | | |

Model 23

| *Predictor* and Covariates | *B* | *SE* | *p-value* | *OR* | 95% *CI* | VIF |
| --- | --- | --- | --- | --- | --- | --- |
| *PIAS negative, peer educator report* | -0.14 | 0.96 | .880 | 0.87 | 0.13-5.67 | 1.05 |
| Available resources | -0.50 | 0.47 | .289 | 0.61 | 0.24-1.53 | 1.19 |
| Access to knowledge | -0.20 | 0.61 | .745 | 0.82 | 0.25-2.72 | 1.18 |
| Leader engagement | -0.65 | 0.46 | .160 | 0.52 | 0.21-1.29 | 1.35 |
| Team climate | -0.44 | 1.14 | .697 | 0.64 | 0.07-5.94 | 1.04 |
| Notes. *B* = unstandardized beta, *SE* = standard error, *OR* = odds ratio, *CI* = confidence interval, VIF = variance inflation factor, PIAS = Provider Intervention Adoption Scale. Likelihood ratio test χ^2^ = 6.17, *p* = .291; Nagelkerke pseudo *R*^2^ = .14 | | | | | | |

Model 24

| *Predictor* and Covariates | *B* | *SE* | *p-value* | *OR* | 95% *CI* | VIF |
| --- | --- | --- | --- | --- | --- | --- |
| *PIAS positive, supervisor report* | 1.07 | 1.37 | .434 | 2.93 | 0.20-43.26 | 1.37 |
| Available resources | -0.55 | 0.47 | .244 | 0.58 | 0.23-1.46 | 1.22 |
| Access to knowledge | -0.07 | 0.63 | .917 | 0.94 | 0.27-3.21 | 1.22 |
| Leader engagement | -0.73 | 0.47 | .119 | 0.48 | 0.20-1.20 | 1.31 |
| Team climate | -0.93 | 1.30 | .474 | 0.40 | 0.03-4.98 | 1.33 |
| Notes. *B* = unstandardized beta, *SE* = standard error, *OR* = odds ratio, *CI* = confidence interval, VIF = variance inflation factor, PIAS = Provider Intervention Adoption Scale. Likelihood ratio test χ^2^ = 6.77, *p* = .239; Nagelkerke pseudo *R*^2^ = .16 | | | | | | |

Model 25

| *Predictor* and Covariates | *B* | *SE* | *p-value* | *OR* | 95% *CI* | VIF |
| --- | --- | --- | --- | --- | --- | --- |
| *PIAS negative, supervisor report* | -0.21 | 1.38 | .877 | 0.81 | 0.50-12.00 | 1.06 |
| Available resources | -0.49 | 0.47 | .295 | 0.61 | 0.24-1.53 | 1.18 |
| Access to knowledge | -0.18 | 0.61 | .771 | 0.84 | 0.25-2.77 | 1.18 |
| Leader engagement | -0.68 | 0.46 | .141 | 0.51 | 0.21-1.25 | 1.32 |
| Team climate | -0.51 | 1.17 | .662 | 0.60 | 0.06-5.89 | 1.07 |
| Notes. *B* = unstandardized beta, *SE* = standard error, *OR* = odds ratio, *CI* = confidence interval, VIF = variance inflation factor, PIAS = Provider Intervention Adoption Scale. Likelihood ratio test χ^2^ = 6.17, *p* = .390; Nagelkerke pseudo *R*^2^ = .14 | | | | | | |

Model 26

| *Predictor* and Covariates | *B* | *SE* | *p-value* | *OR* | 95% *CI* | VIF |
| --- | --- | --- | --- | --- | --- | --- |
| *Modified Practice Attitudes Scale* | 0.29 | 1.60 | .854 | 1.34 | 0.59-30.67 | 1.17 |
| Available resources | -0.49 | 0.47 | .295 | 0.61 | 0.24-1.54 | 1.18 |
| Access to knowledge | -0.20 | 0.61 | .740 | 0.82 | 0.25-2.72 | 1.20 |
| Leader engagement | -0.64 | 0.48 | .180 | 0.53 | 0.21-1.34 | 1.44 |
| Team climate | -0.50 | 1.14 | .665 | 0.61 | 0.07-5.74 | 1.06 |
| Notes. *B* = unstandardized beta, *SE* = standard error, *OR* = odds ratio, *CI* = confidence interval, VIF = variance inflation factor. Likelihood ratio test χ^2^ = 6.18, *p* = .289; Nagelkerke pseudo *R*^2^ = .14 | | | | | | |

Model 27

| *Predictor* and Covariates | *B* | *SE* | *p-value* | *OR* | 95% *CI* | VIF |
| --- | --- | --- | --- | --- | --- | --- |
| *PIPS number of implementation activities* | 0.17 | 0.13 | .179 | 1.19 | 0.93-1.52 | 1.09 |
| Available resources | -0.42 | 0.49 | .388 | 0.66 | 0.25-1.70 | 1.19 |
| Access to knowledge | -0.32 | 0.63 | .609 | 0.73 | 0.21-2.47 | 1.19 |
| Leader engagement | -0.68 | 0.47 | .147 | 0.51 | 0.20-1.27 | 1.30 |
| Team climate | -0.93 | 1.23 | .449 | 0.40 | 0.04-4.38 | 1.09 |
| Notes. *B* = unstandardized beta, *SE* = standard error, *OR* = odds ratio, *CI* = confidence interval, VIF = variance inflation factor, PIPS = Prevention Implementation Progress Scale. Likelihood ratio test χ^2^ = 8.05, *p* = .153; Nagelkerke pseudo *R*^2^ = .19 | | | | | | |
